# Dairy Goats’ Responses to Different Levels of Pomegranate By-Product Silage in the Diet: Productive Performance and Metabolic Indicators

**DOI:** 10.3390/ani16162456

**Published:** 2026-08-07

**Authors:** José Ramón Díaz, Marina Galvez-Lopez, Dieu donné Kiatti, Amparo Roca, Mihaela Iasmina Madalina Ilea, Alberto S. Atzori, Gema Romero

**Affiliations:** 1 Institute of Agri-Food and Agro-Environmental Research (CIAGRO-UMH), Miguel Hernández University of Elche, Carretera De Beniel, km 3.2, 03312 Orihuela, Spain; jr.diaz@umh.es (J.R.D.); mihaela.ilea@goumh.umh.es (M.I.M.I.); gemaromero@umh.es (G.R.); 2 Department of Agricultural Science, University of Sassari, Viale Italia 39, 07100 Sassari, Italy; kiattidieudoone@gmail.com (D.d.K.); asatzori@uniss.it (A.S.A.); 3 Technical Support Service for Teaching and Research (SATDI-UMH), Miguel Hernández University of Elche, Carretera De Beniel, km 3.2, 03312 Orihuela, Spain; aroca@umh.es; 4 Institute for Animal Production System in Mediterranean Environment, National Research Council (CNE-ISPAAM), 07100 Sassari, Italy

**Keywords:** agrifood by-product, circular economy, animal nutrition, oxidative stress, biochemical profile

## Abstract

The current study evaluated the effects of different levels of inclusion of ensiled pomegranate by-product (PS: 0, 5, 10, and 15% DM) into the diet of lactating dairy goats on production performance and metabolic status. Seventy-two Murciano-Granadina goats were used and balanced split into the four treatments (18 goats/treatment × 4 treatments) and also distributed into two homogeneous batches per treatment to record diet and water ingestion at batch level, according to individual variables (milk yield, body weight, SCC, and parity) obtained in a pre-experimental sampling, when all the variables at the individual (milk yield and composition, body weight, blood) and batch level (DM and water intake) were recorded. Later, the feeding trial was started and conducted for 8 weeks, with body weight, milk yield, and milk composition—fat, crude protein, lactose, milk urea, somatic cell count (SCC)—recorded biweekly. Water intake (WI) and dry matter intake (DMI) were registered weekly over 2 consecutive days. Physiological and oxidative status were reviewed through hemogram, serum biochemical profile, and plasma antioxidant activity at the last sampling. Individual level variables were analyzed using mixed linear models with repeated measures, including diet, sampling, and their interaction as fixed effects, the covariate in the pre-experimental sampling, and the animal and the batch nested to diet as the random effects (PROC GLIMMIX, SAS v9.4). Additionally, observed means and standard deviations of WI and DMI were recorded. The results showed that milk yield and its composition were not significantly (*p* > 0.05) affected by up to 15 PS inclusion. The statistical differences observed in the hematological and plasma biochemical profiles, as well as in the antioxidant activity, had no practical relevance, as all measured values remained within the established physiological reference ranges. In conclusion, PS can be added to dairy goats diet up to 15% with no evidence of negative effects on production and animal physio-metabolic response during the study period, although longer trials considering complete lactation periods are needed to corroborate these findings.

## Simple Summary

This study provides practical insight into the use of pomegranate by-product silage as an alternative feed ingredient for dairy goats. Ensiling allows the incorporation of substantially higher inclusion levels than those typically achievable with meals or extracts, offering a more effective valorization pathway for an abundant agro-industrial residue. By integrating this by-product into goat diets, the approach contributes to circular-economy strategies and may support more sustainable feeding systems in a sector experiencing growing global demand for milk and dairy products.

## 1. Introduction

The sustained growth of the world population and evolving consumer dietary preferences have led to a steady expansion of the food production and manufacturing industry [1]. However, the livestock industry—strongly dependent on cultivated cereals, legumes, and traditional forage crops—is experiencing a significant decline in competitiveness [2]. Concurrently, substantial food losses occur throughout the supply chain, from harvest to final processing, amounting to an estimated 1.3 billion tons annually, of which approximately 60% corresponds to fruits and vegetables [3]. Within the European Union, recent assessments indicate that around 37 million tons of agro-industrial by-products (peels, stems, seeds, and pulp) are generated each year [4]. The incorporation of agro-industrial by-products in animal feed supports circular economy goals by reducing feed costs, recycling nutrients, and enhancing product quality through bioactive compounds [5,6]. Tunisia, Turkey, Israel, and Spain currently rank among the top global producers of pomegranate (*Punica granatum* L.), leading in terms of cultivated area, fruit yield, and export volume [7]. In Spain, pomegranates are predominantly processed for juice, resulting in substantial quantities of by-products, particularly peel and seeds, which are notably rich in polyphenols, such as phenolic acids, tannins, flavonoids, and anthocyanins [8]. These compounds are responsible for its pronounced antioxidant capacity [9,10], contributing to reduced risk of cardiovascular disease and enhanced cellular longevity [11,12]. Owing to this bioactive richness, pomegranate peel has gained increasing attention as a functional ingredient in both human and animal nutrition. Ruminants are especially adapted to a fibre-rich diet and bioactive compounds with sustained digestive availability, common characteristics of plant-based by-products [13,14]. These animals play a crucial role in closing nutrient cycles within circular food systems by transforming low-opportunity-cost biomass into high-quality meat and milk, both of which provide essential and well-balanced amino acids [15].

Numerous studies have examined the use of pomegranate by-products as dietary supplements for ruminants [16,17]. It has been observed that the ensiling process is an effective method for preserving the antioxidant integrity of peel and seeds [18,19]. Jami et al. [20] observed that including up to 4% concentrated pomegranate extract in dairy cow diets enhanced milk production, increased dry matter intake, and improved nutrient digestibility. Moreover, tannins derived from peel can decrease ruminally protein breakdown, thereby improving the amount of protein that becomes available post-ruminally [14,20]. More recently, in vitro investigations have confirmed that both seeds and peel are easily fermented by rumen microorganisms, indicating promising potential as a feedstock for ruminant diets [16]. Ahmed et al. [17] observed that incorporating pomegranate peel with molasses and berseem clover silage not only improved fermentation characteristics but also lowered methane emissions in buffaloes, likely due to tannin inhibition of methanogenic archaea. Zhang et al. [18] further attributed reductions in nitrogen losses and methane emissions to the antioxidant properties of the pomegranate peel. In cattle studies, such as those by Eliyahu et al. [21] and Kazemi et al. [22], the inclusion of pomegranate by-product in total mixed rations yielded positive outcomes, improving feed intake, nutrient degradability, growth performance, and meat quality in fat-tailed lambs and sheep, respectively, when pomegranate by-product silage was incorporated into total mixed rations. However, the caprine species has been scarcely evaluated to date despite presenting a distinct physiological advantage. Goats exhibit a higher tolerance to tannins than sheep or cattle, a characteristic linked to salivary proteins with a strong tannin-binding capacity [23]. This adaptive mechanism may mitigate the dose-dependent antinutritional effects reported in other species when high levels of tannins are administered [24].

In this context, the present research aimed to evaluate the impact of including varying levels of pomegranate silage into the diets of lactating dairy goats on individual productive performance and gross composition—including somatic cell count—as well as on body weight, physiology and metabolic response, and oxidative stress biomarkers. The ultimate goal was to identify the optimal inclusion level from both nutritional and environmental standpoints, suitable for integration into dairy goat feeding strategies.

## 2. Materials and Methods

### 2.1. Animals and Facilities

The experiment was conducted using the dairy goats maintained at the research facilities of Miguel Hernández University (Spain). The trial extended from mid-September—when the pre-experimental screening took place—until the start of November. The herd, composed of Murciano-Granadina goats, was managed under free-stall housing with straw bedding. Each animal was allocated in 1.5 m^2^ of individual area, provided with 35 cm of linear feeding space, and had constant access to water. Feeding was appointed twice daily, at 9:00 and 15:00, and milking was performed once per day using a parallel milking parlour (2 × 12 × 12 configuration, GEA, Düsseldorf, Germany), following standard regional practices. The study received ethical approval from the Office of Responsible Research at Miguel Hernández University (approval code: UMH.DTA.JDS.02.21).

### 2.2. Experimental Design

From an initial herd of 100 goats (at approximately 60 ± 4 days postpartum), and feeding a standard lactating goat’s diet, 72 animals were selected for the study. Selection was based on parity, body weight (40.35 ± 1.70 kg), milk yield (2.16 ± 0.15 kg/day), and LSCC (2.75 ± 0.12 103 cells/mL), all measured at individual level. The goats were assigned to the four treatments and split into eight homogeneous batches (two batches/treatment) to record diet and water ingestion at batch level, ensuring balanced distribution of the batches across the selection criteria. After a 1-week adaptation period to their newly assigned groups, a pre-experimental sampling (sampling 0) was conducted, in which all variables under evaluation were measured. Afterward, the feeding trial started, and the four experimental diets were randomly allocated to two batches each:

- Control: conventional diet without by-products,
- PS_5: conventional diet including 5% of ensiled pomegranate by-product (PS) on DM basis,
- PS_10: diet including 10% DM of PS,
- PS_15: diet including 15% DM of PS.

Following a 2-week feed adaptation period, 4 biweekly samplings were conducted to evaluate individual body weight, milk yield, and its gross composition. Only one more individual blood sample was collected at the end of the experiment. Water and feed intake were registered weekly for 2 consecutive days at the batch level.

Ration feeds were formulated in accordance with the guidelines established by Fernández et al. [25] for dairy goats yielding approximately 2.0 kg of milk per day, ensuring that all rations were isoenergetic and isoproteic and matched to the animals’ productive requirements. Feed was provided in fixed quantities twice daily without ad libitum access. All ingredients were offered at the same time as separate components distributed along the linear feeder. Comprehensive information on the ingredient composition, chemical profile, and daily feed allocation is presented in Table 1. The Pomegranate silage incorporated into the experimental diets consisted of a mixture of 80% pomegranate by-product, variety Wonderful, and 20% cereal straw. This pomegranate silage had been produced 6 months earlier and originated from the quality and stability trial reported by Galvez-Lopez et al. [19].

### 2.3. Analysed Variables

The nutritional profile of the diets (Table 1) was determined per duplicated on a sample of every diet, using standardised methods of AOAC [27]: organic matter (OM, method 942.05); dry matter (DM, method 930.5), crude protein (CP, method 984.13); ether extract (EE, method 920.39); and total sugars (method 974.06). The fibre fractions included neutral detergent fibre (NDF, g/kg DM), acid detergent fibre (ADF, g/kg DM), and acid detergent lignin (ADL, g/kg DM), and they were determined by Van Soest et al. [28]. Starch content was measured using Ewers polarimetric method [29].

Total polyphenol content (PT, g GAE eq./kg DM) was determined according to the Folin-Ciocalteu colourimetric method following the specifications of Kim et al. [30]. The mineral profile was identified through acid digestion of diet samples, using an Inductively Coupled Plasma Mass Spectrometer (ICP-MS; Agilent 7700x, Aligent, Santa Clara, CA, USA), according to González Arrojo et al. [31]. Moreover, apparent in vitro dry matter digestibility (IVDMD, g/kg DM) was estimated using the method of Menke and Steingass [32], with slow modifications by Yáñez-Ruiz et al. [33]. To evaluate diet antioxidant potential, ABTS (2,2′-azino-bis (3-ethylbenzothiazoline-6-sulfonate radical) and DPPH (1,1-diphenyl-2-picryl-hydrazyl radical) reducing power were determined. The ABTS assay followed the method described by Leite et al. [34], while the DPPH assay was conducted following the revised protocol by Cheng et al. [35], developed initially by Brand-Williams et al. [36]. Final absorbance for these assays was recorded using the UV-VISIBLE spectrophotometer (Zuzi 4255/50, Foss, Hillerød, Denmark at wavelengths of 515 and 734 nm, for DPPH and ABTS, respectively. The results were expressed as Trolox equivalents (mg Trolox eq/g DM).

At each sampling point, all goats were weighed using a high-precision scale (Accurex RX Fox, Gram Group, Barcelona, Spain) and integrated into the handling chute, with a measurement accuracy of 100 g to monitor body weight (kg) progression throughout the trial. Daily individual milk yield (kg/day) was registered using a Lactocorder^®^ device (Lactocorder, Balgach, Switzerland). Milk macrocomposition—including crude protein, lactose, fat, non-fat dry matter (NFDM), useful dry matter (UDM), and urea content—was determined by mid-infrared spectroscopy (CombiFoss™ 7 DC, Foss, Hillerød, Denmark). Somatic cell count (SCC, 10^3^ cells/mL) was assessed via flow cytometry using the same equipment. Energy-Corrected Milk (ECM [37] (1)) and Fat and Protein-Corrected Milk (FPCM, kg/day) were calculated according to Schau and Fet [38] (2):

ECM = milk yield (kg/day) × ((38.3 × fat (g/kg) + (24.2 × crude protein (g/kg) + (16.54 × lactose (g/kg) + 20.7)/3140(1)

FPCM = milk yield (kg/day) × (0.337 + 0.116 × fat (%) + 0.06 × crude protein (%)) (2)

Water intake (WI) and dry matter intake (DMI) were registered weekly at the batch level over 2 consecutive days during each sampling week. Feed intake was estimated as the difference between the quantity of diet offered and the amount refused, on a fresh and dry matter basis. Dry matter content was determined by oven-drying representative samples of the offered and refused diets at 105 °C for 48 h. Water intake was calculated by difference using water meters (Fenix Hidroconta-SV-RTK E4^®^ Flow Systems, Murcia, Spain) placed in each pen. Data/animal and day were calculated for WI (L/day) and DMI (kg/day), dividing by the number of goats in the batch.

Blood samples fasted for 12 h were collected from 72 goats (18 goats/treatment, 2 batches/treatment) only at baseline and final experimental timing. Blood was collected from the jugular vein using an Eclipse™ needle (BD Vacutainer, Franklin Lakes, NJ, USA) and preserved in three test tubes (BD Vacutainer, Franklin Lakes, NJ, USA): EDTA K2 (4 mL) for hematological analysis, Serum CAT (10 mL) with clot activator for serum biochemical profile assessment, and LH Lithium Heparin (10 mL) for oxidative status evaluation. Centrifugation of the samples collected in serum CAT and LH tubes was performed immediately after blood collection, and the resulting serum and plasma were stored at −80 °C and analysed within 1 month of sampling. Haematology was analysed using an Element HT5 analyser (SCIL VET, Tudela, Spain) calibrated for a female goat: white blood cells (WBC), monocytes (MON), lymphocytes (LYM), neutrophils (NEU) and the ratios monocytes/lymphocytes (MLR), and neutrophils/lymphocytes (NLR) were calculated regarding the leukogram. Red blood cell count was determined based on the following erythrocyte indices: red blood cells (RBC), hematocrit (HCT), haemoglobin concentration (HGB), mean corpuscular volume (MCV), mean corpuscular haemoglobin concentration (MCHC), mean corpuscular haemoglobin (MCH), and red cell distribution width—coefficient of variation (RDW-CV). Serum biochemical profiling was determined using the rapid diagnostic disk “Diagnostic II” for Element RC3X (SCIL VET, Tudela, Spain). The variables analyzed included: thyroxine (T4), alkaline phosphatase (ALP), cholesterol (CHOL), glucose (GLU), albumin (ALB), amylase (AMY), protein (TP), blood urea nitrogen (BUN), alanine aminotransferase (ALT), total bilirubin (TBIL), creatinine (CREA), globulin (GLOB), Na, Ca, K, and P. Oxidative stress was determined using total antioxidant capacity assay—FRAP Oyaizu [39] and ABTS Leite et al. [34]. Specific antioxidant capacity was evaluated using superoxide dismutase (SOD) activity assay (DetectX^®^ Superoxide Dismutase Activity Kit, Arbor Assays, Ann Arbor, MI, USA).

### 2.4. Statistical Analysis

The SCC values underwent base-10 logarithm transformation before statistical analysis. Individual data (excluding blood parameters) were calculated using a mixed linear model (PROC GLIMMIX, SAS v9.4), considering the effect of the pre-experimental covariate, diet, samplings, the interaction (diet × sampling), and batch (diet), following the next Equation (3):

Y = µ + D_i_ + S_j_ + D_i_ × S_j_ + covY_0_ + B_k_(Di) + A_k_ + e (3)

where Y is the dependent variable, µ is the intercept, D_i_ denotes the fixed effect of diet (i = Control, PS-5, PS-10, PS-15), S_j_ is the fixed effect of sampling (j = 1, 2, 3, 4), D_i_ × S_j_ is the interaction between diet and sampling (16 levels), covY_0_ accounts for the effect of Y in the pre-experimental sampling, B_k_(Di) is the random effect of batch nested within diet, and e is the residual error. The animal was always considered a random effect (A_k_). An autoregressive (AR1) covariance structure was selected for repeated measures, based on the lowest AIC and BIC values.

For blood samples, the model included the fixed effects of diet (i = four levels), sampling (two levels, j = 0, 4), their interaction (eight levels), and the random effect of batch (diet). The animal was always considered a random effect (A_k_). A compound symmetry (CS) covariance structure was selected for repeated measures, based on the lowest AIC and BIC values.

In the mixed models, the variance of the random effect batch (diet) was estimated as zero for the selection-balancing variables, which shifted the corresponding tests to the animal level with higher denominator degrees of freedom, whereas the remaining variables retained ≈4 denominator degrees of freedom (*df*).

Least-squares means comparison (Tukey test) was used to evaluate differences among levels of the fixed effects. Statistical significance was established at *p* < 0.05, leading to the rejection of the null hypothesis and confirming meaningful differences between factor levels.

For batch-level data (WI, DMI), observed means and standard deviation were calculated for every treatment and sampling.

## 3. Results

**Figure 1:**
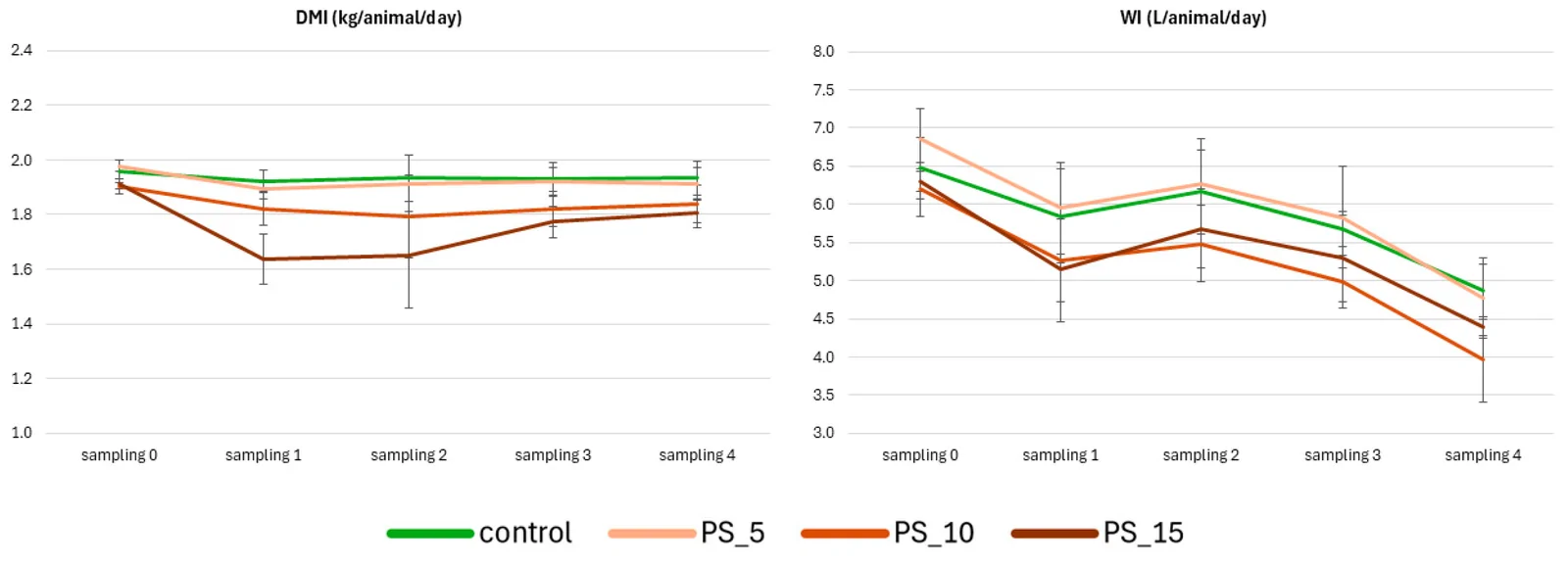
Observed means ± standard deviation of dry matter intake (DMI) and water intake (WI) of dairy goats fed the four experimental diets throughout the study period. PS: pomegranate silage; PS_5: experimental diet with 5% of PS; PS_10: experimental diet with 10% of PS; PS_15: experimental diet with 15% of PS. n = eight batches (two batches/treatment, with nine goats/batch) for DMI and WI.

Dry matter intake (DMI, observed mean ± standard deviation) showed a gradual decrease with the inclusion of PS, falling from 1.94 ± 0.06 kg/day in the control group to 1.92 ± 0.06 kg/day in PS_5, 1.84 ± 0.09 kg/day in PS_10, and 1.76 ± 0.14 kg/day in PS_15. Water intake (WI, observed mean ± standard deviation) showed moderate variations among treatments, with means of 5.81 ± 0.69 L/day (control), 5.69 ± 0.28 L/day (PS_5), 5.17 ± 0.83 L/day (PS_10), and 5.36 ± 0.77 L/day (PS_15). The evolution throughout experimental time is presented in Figure 1.

**Figure 2:**
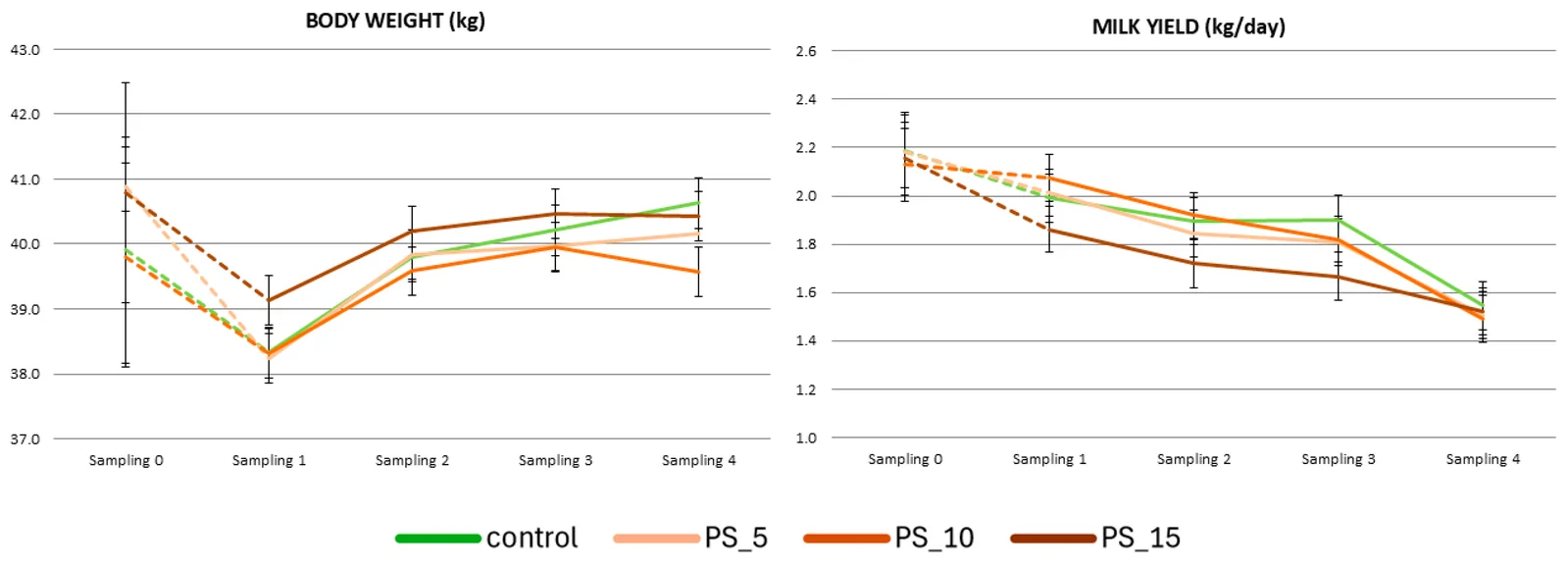
Effect of the inclusion of pomegranate silage on body weight and milk yield (estimated means and SE bars according to Tukey test) of dairy goats throughout the sampling period. Dotted lines correspond with the pre-experimental period. PS: pomegranate silage; PS_5: experimental diet with 5% of PS; PS_10: experimental diet with 10% of PS; PS_15: experimental diet with 15% of PS. n = 72 animals (18 goats/treatment, 2 batches/treatment).

The statistical effect of diets containing pomegranate silage on productive performance traits of dairy goats is presented in Table 2. The inclusion of pomegranate silage in dairy goats’ diets maintained without statistical differences in body weight (BW) and milk yield (MY). Regarding milk yield, although not significant, it was observed to have a lower numerical value at PS_15 diet (1.69 kg/day vs. 1.73–1.83 kg/day in the other diets, SE: 0.07). However, not all diets affected the milk composition and milk correction indexes (ECM and FPCM). On the other hand, all the parameters analysed were significantly different during the sampling, but the interaction between diet and sampling effects was not significantly different. Evolutionary trends of variables showing significant treatment effects across the successive samplings are represented in Figure 2. At the fourth sampling, the parameters were similar except for PS_10 for BW, which was lower compared to the control diet (Figure 2). Concerning MY, a significant decrease was observed in all four diets between sampling 0 and sampling 4.

The effect of the inclusion of PS in the dairy goats’ diet on blood haematological traits is presented in Table 3. The data were reported at the beginning (sampling 0) and at the end (sampling 4) of the experiment. The diets did not affect the goats’ blood haematological traits (*p* > 0.05). Variations along the samplings were observed for red blood cells (RBC), haemoglobin (HGB), haematocrit (HCT), mean corpuscular volume (MCV), and mean corpuscular haemoglobin concentration (MCHC) and red blood cell distribution width coefficient of variation (RDW-CV). Sampling 4 showed the highest values for all these affected parameters except the MCV, where the highest was observed at sampling 0. In addition, the highest and lowest values were observed in the animals fed with the control and PS_15 diet, respectively, while the animals fed with PS_5 and PS_10 diet were intermediate, even though significant differences were not always observed.

Furthermore, pomegranate silage did not affect the plasma biochemical profile of the goats (Table 4). However, alanine aminotransferase (ALT), thyroid hormone (T4), total Bilirubin (TBIL), creatinine (CREA), blood urea nitrogen (BUN), phosphorus (P), glucose (GLU), sodium (Na), total protein (TP), albumin (ALB), and globulin (GLOB) varied within the sampling period (*p* < 0.05). In general, the highest values of these parameters were observed at sampling 4, while only P and K showed the highest at sampling 0.

The effect of diet containing pomegranate silage on plasma total and antioxidant capacity of dairy goats is reported in Table 5. The diet did not significantly affect the plasma antioxidant capacity of dairy goats, but 2,2′-azino-bis (3-ethylbenzothiazoline-6-sulfonate) (ABTS) and ferric reducing antioxidant power (FRAP) varied significantly from sampling 0 to sampling 4. ABTS measured at sampling 0 was higher than the one measured at sampling 4, whereas the opposite finding was observed for FRAP. In general, the interaction between diet and sampling was not significant throughout the study.

## 4. Discussion

### 4.1. Effect of Pomegranate Silage on Intake, Body Weight, Milk Yield, and Milk Composition

The unchanged milk yield and gross composition, together with the stable body weight observed across groups despite the differences in diet composition and the distinct DMI patterns, can be attributed to homeorhesis—the coordinated physiological regulation that prioritizes nutrient partitioning to sustain the dominant metabolic functions in dairy ruminants [40]. This response further highlights the strong adaptability of the Murciano-Granadina breed to intensive milk-production systems. In early lactation, homeorhesis strongly drives nutrients toward milk production, maintaining productive stability and buffering moderate dietary variations. Homeorhesis ensures a coordinated and consistent flow of nutrients to sustain dominant physiological states such as lactation [41]. The changes observed in the fibre fractions could, in principle, influence rumen fill and chewing activity and thereby contribute to differences in DMI, since higher fibre fractions (particularly NDF and ADF) increase rumen retention time and stimulate chewing and rumination, which are well-known determinants of voluntary intake in ruminants [42,43]. However, the range of variation in NDF and ADF among treatments was relatively narrow, and all diets remained within typical values for well-formulated dairy goat rations [25]. Correddu et al. [44] similarly reported no changes in milk yield or composition in small dairy ruminant-fed diets containing comparable levels of pomegranate by-products (70 to 120 g/kg DMI). Other authors testing pomegranate silage at different concentrations varying from 12% to 24% in small ruminants’ total mixed ration did not find any effect on DMI [21,45]. For example, Kotsampasia et al. [45] did not observe differences for DMI and feed conversion ratio within the treatments (0, 120, and 240 g/kg DM) when they tested the effects of dietary pomegranate by-product silage on performance, carcass characteristics, meat chemical, and fatty acid composition of growing Florina (Pelagonia) male lambs. Moreover, several studies evaluating pomegranate by-products—either dried or ensiled—in ruminant nutrition have reported results consistent with the present findings, showing no changes in body weight, milk yield, or milk composition. Valenti et al. [46] reported no changes in milk yield or composition when 64.8% pomegranate pulp was included in the concentrate of late-lactation grazing ewes. Likewise, other authors have shown that incorporating pomegranate by-products at levels ranging from 5% to 40% of DM generally does not affect milk yield or the fat and protein content of dairy ruminants [44,47]. Regarding the progress of the variables along the experiment, the decrease observed in Figure 2 for BW and MY for all the treatments is explained by the normal lactation curve after reaching the peak and the progressive adaptation to the diets, which contained a higher inclusion of PS that the animals were not familiarised with. The pattern observed in milk composition agrees with the normal evolution at the same stage of lactation for the same breed, as the milk yield, lactose, and urea decrease after reaching the peak, while fat and UDM increase [48]. In general, the observed patterns were consistent with the physiological evolution of lactation rather than with treatment effects. Differences in the composition of pomegranate by-products (pulp, seed, or peel) and in the form in which they are incorporated into animal diets (dried or ensiled) may account for the variation between our findings and previous reports. Correddu et al. [44] confirmed that pomegranate seed, pulp, and peel provide different amounts of ether extract, neutral detergent fibre (NDF), and probably total polyphenols in ruminant TMR diet, which animals could respond differently in terms of intake, rumen fermentation, and volatile fatty acids production.

### 4.2. Effect of Pomegranate Silage on Blood Haematological Traits, Plasma Biochemical, and Plasma Antioxidant Capacity of Dairy Goats

Feeding pomegranate silage to dairy goats up to 15% of DM showed no effect on blood haematological traits and plasma biochemicals, so no adverse effects on animals’ status at the tested levels; similar findings were reported by Obeidat [49]. These parameters serve as integrated indicators of how the animals were supplied with nutrients present in the formulated diet, which, in our case, contained pomegranate silage. The haematological profile and serum biochemistry measured throughout the study remained within the established physiological reference ranges for dairy goats [50]. Packed cell volume (22–38%) and haemoglobin concentrations (8–12 g/dL) were within the limits reported for healthy goats, indicating adequate erythropoietic status and the absence of anaemia or haemoconcentration [50,51]. Total leukocyte counts (4–13 × 10^9^/L) and the differential profile also remained normal, with neutrophils and lymphocytes fluctuating within their typical physiological ranges (20–50% and 50–70%, respectively), suggesting that no physiological change was triggered by the dietary treatments [51,52]. Likewise, key biochemical indicators of metabolic and hepatic function, including glucose (2.2–4.2 mmol/L), blood urea nitrogen (3.6–16.1 mmol/L), creatinine (44–150 µmol/L), ALT (12–40 U/L), total bilirubin (1.7–8.6 µmol/L), and cholesterol (1.6–3.4 mmol/L), remained stable and within reference intervals, confirming the absence of metabolic distress or liver dysfunction [50,53,54]. Finally, thyroid hormone concentrations (T4: 4–10 µg/dL) remained within normal physiological ranges, indicating that energy balance and endocrine function were not adversely affected by the inclusion of the experimental silages [55]. Taken together, these results show that dietary treatments did not compromise the animals’ physiological homeostasis. Klinkon and Ježek [52] reported that the feeding and rearing system can induce variations in blood haematological characteristics and plasma biochemical compounds. In addition, Ramzi [56] found no changes in key haematological, biochemical, or endocrine indicators when pomegranate peel was included up to 4% in lambs diets, a response that aligns with the results obtained in the current study. Moreover, Bakr et al. [57] did not observe any effect on blood parameters (ALT, AST, cholesterol, triglyceride, urea, creatinine, albumin, and total protein) of heifers fed with dried pomegranate peel at 100 and 200 g/h/d, similar to the results reported in dairy animals by Akhlaghi et al. [58]. Regarding the effect of progress of lactation, we observed an increase of RBC, HGB, haematocrit, MCV, MCHC, T4, ALT, total Bilirubin, blood urea nitrogen, creatinine, glucose, sodium, total protein, albumin, and globulin from sampling 0 to sampling 4, but still in the normal range reported for small ruminants [51,53]. The increase in RBC and T4 observed in both the PS treatment and the control group at the final sampling point can be explained by the concurrent rise in body weight and the natural progression of the lactation stage (Figure 1). The same results were reported by Soca et al. [54] and Todini et al. [55], evaluating factors affecting haematological parameters and plasma total T3 and T4 concentrations in goats, respectively. Al-Musodi and Al-Zwean [59] observed a significant increase in haematological and immune parameters in lambs after 3 months of feeding them pomegranate seed powder up to 6%, attributing these results to the presence of important nutrients such as iron, zinc, copper, vitamins, flavonoids, and tannins in pomegranate seeds, which promote the production of haematological elements. In dairy cows, Safari et al. [60] showed that the supplementation with pomegranate by-products (i.e., seed: 400 g/cow per day and seed + peel: 400 g of seeds/cow + 1200 g of peels/cow per day) did not affect some plasma biochemicals (cholesterol, total protein, and globulin) at prepartum and postpartum periods but reduce blood glucose concentration, probably due to the increase of insulin during the earlier period of postpartum compared with the control group. Overall, the current study and the previously published confirm that pomegranate by-products could be used in ruminants’ nutrition without a negative effect on animal metabolic status.

On the other hand, the inclusion of pomegranate silage at 5%, 10%, and 15% of DM in dairy goats’ diet did not significant change the plasma antioxidant capacity (ABTS, FRAP and SOD) and could be due to the short experimental time and moderate incorporation levels that limited an immediate functional response with a physiological effect on systemic antioxidant capacity, even the potential presence of bioactive compounds in PS. These results are opposite to those reported by Kotsampasi et al. [45] for FRAP and ABTS, which measure the total antioxidant activity and free radical scavenging activity, while SOD activity linearly increased with pomegranate pulp silage inclusion at 75 and 150 g/kg of DM in the TMR of lactating dairy cows. The authors attributed the increase in SOD to the high antioxidant properties of pomegranate by-products due to the presence of phenolic compounds, mainly punicalagin and ellagitannins. Moreover, silage treated with pomegranate by-products showed increased flavonoid, total phenol, and polysaccharide contents, likely to reflect the naturally high concentrations of these compounds in pomegranate by-products [18,61] and may consequently enhance the antioxidant capacity of ruminants [62,63,64]. However, the unaffected plasma antioxidant capacity in this study could be explained by several factors, such as the short length of the trial, the moderate inclusion levels of the by-product, the effect of polyphenols on rumen microorganism activities in carbohydrate and protein digestion, and intestinal absorption. Vasta et al. [65] reported that low or moderate incorporation of polyphenols from processing by-products in the diet of dairy ruminants did not affect production performance and animals’ status parameters. Despite this, several authors have reported beneficial effects of the bioactive compounds present in pomegranate processing by-products, including reductions in ruminal protozoa counts, ammonia–nitrogen concentrations, protein degradation, and gas and methane production [18,64].

## 5. Conclusions

The results indicate that the incorporation of up to 15% DM of pomegranate by-product silage in isoenergetic and isoproteic diets for dairy goats maintained milk yield, milk quality, and body weight, with no evidence of negative effects on the animals’ status (biochemical, inflammatory, oxidative stress) at the tested sample size and trial length. These findings support the use of pomegranate silage, at the tested inclusion levels, as a viable feed resource from a productive perspective, giving an alternative that may increase the environmental sustainability of the dairy goat industry. Given the presence of tannins and other phenolic compounds in this by-product, long-term studies covering at least a full lactation period are required to confirm that prolonged consumption does not compromise milk yield, animal status, ruminal metabolism, or other productive performance, and to further validate its practical applicability under commercial conditions.

## Figures and Tables

**Table 1:** Ingredients and chemical composition of experimental diets containing different inclusions of pomegranate silage for feeding dairy goats.

Item	Diets			
	Control	PS_5	PS_10	PS_15
***Ingredients (% DM)***				
Pomegranate/Straw Silage (80/20)	-	6.1	12.3	18.4
Alfalfa hay (16% CP)	29.4	22.0	13.4	13.5
Barley straw	7.6	11.1	11.5	7.1
Soybean cake (44% CP)	-	4.2	6.2	6.4
Medium-ground Corn	-	3.2	2.9	1.0
Pelleted grain mix	63.0	53.6	53.6	53.6
kg DM offered/animal/day	2.1	2.1	2.1	2.0
***Chemical composition (g/kg DM)***				
DM (g/kg FM)	923.3	820.7	753.2	678.3
OM	852.7	859.4	864.7	866.7
EE	33.0	26.0	32.0	31.0
CP	186.0	186.0	184.0	184.0
NDF	395.0	378.0	361.0	364.0
ADF	228.0	221.0	195.0	198.0
ADL	34.0	36.0	25.0	26.0
Starch	190.0	204.0	218.0	204.0
Total Sugar	27.0	28.0	30.0	27.0
IVDMD	699.7	718.9	724.1	718.0
ME ^1^ (Mcal/kg DM)	2.1	2.2	2.2	2.2
MFU	1.9	1.9	1.9	1.9
Total phenols (mg GAE eq./g DM)	12.4	15.5	17.1	18.0
***Mineral profile (g/kg DM)***				
Ca	13.0	11.0	11.0	11.0
P	4.0	4.0	4.0	4.0
Na	5.0	4.0	4.0	4.0
K	16.0	16.0	14.0	16.0
Mg	3.0	3.0	3.0	3.0
***Antioxidant activity (mg Trolox eq/g DM)***				
ABTS	2.3	2.7	2.9	3.1
DPPH	0.8	0.9	1.4	1.5

PS_5. experimental diet with 5% of PS; PS_10: experimental diet with 10% of PS; PS_15: experimental diet with 15% of PS; DM: dry matter; FM: fresh matter; OM: organic matter; CP: crude protein; EE: ether extract; NDF: neutral detergent fiber; ADF: acid detergent fiber; ADL: acid detergent lignin; IVDMD: in vitro dry matter digestibility; ME: Metabolizable energy: ^1^ Jarrige et al. [26]; MFU: milk forage unit; DPPH: 1,1-Diphenyl-2-picryl-hydrazyl; ABTS: 2,2′-azino-bis (3-ethylbenzothiazoline-6-sulfonate).

**Table 2:** Effect of diets containing pomegranate silage on productive performance traits of dairy goats.

Item	Diets					*p*-*Value*		
	Control	PS_5	PS_10	PS_15	SEM	Diet	Sampling	Diet × Sampling
Body weight (kg)	39.74	39.56	39.35	40.06	0.33	0.486	<0.001	0.221
Milk yield (kg/d)	1.83	1.79	1.83	1.69	0.07	0.506	<0.001	0.835
**Milk composition**								
Fat (%)	4.76	4.87	4.83	4.92	0.24	0.970	<0.001	0.377
Protein (%)	3.78	3.75	3.73	3.69	0.05	0.674	<0.001	0.841
Lactose (%)	4.64	4.63	4.63	4.59	0.03	0.665	<0.001	0.431
NFDM (%)	9.25	9.27	9.19	9.18	0.06	0.673	<0.001	0.482
UDM (%)	8.52	8.63	8.57	8.62	0.29	0.992	<0.001	0.397
Urea (mg/L)	566	566	554	568	11	0.800	<0.001	0.733
LSCC (Log10^3^ cell/mL)	2.75	2.89	2.73	2.76	0.09	0.595	<0.001	0.300
**Milk correction indexes**								
FPCM (kg/d)	1.96	1.97	1.98	1.88	0.09	0.858	<0.0001	0.302
ECM (kg/d)	1.98	1.99	1.98	1.88	0.09	0.863	0.0002	0.294

PS_5: experimental diet with 5% of PS; PS_10: experimental diet with 10% of PS; PS_15: experimental diet with 15% of PS; SEM: standard error of the mean; NFDM: non-fat dry matter; UDM: useful dry matter (% fat + % protein); LSCC: log10 somatic cell count; FPCM: fat and protein corrected milk; ECM: energy corrected milk.

**Table 3:** Effect of diets containing pomegranate silage on blood haematological traits of dairy goats.

Item		Diets					*p*-*Value*		
		Control	PS_5	PS_10	PS_15	SEM	Diet	Sampling	Diet × Sampling
**WBC (10^9^/L)**	*(Sampling 0)*	15.07	12.80	14.10	12.26	1.17	0.356	0.694	0.677
	*(Sampling 4)*	14.63	13.49	14.93	11.92	1.14			
**LYM (10^9^/L)**	*(Sampling 0)*	4.61	3.83	3.87	4.06	0.96	0.274	0.431	0.223
	*(Sampling 4)*	4.31	4.13	4.59	3.80	0.98			
**MON (10^9^/L)**	*(Sampling 0)*	0.19	0.16	0.12	0.13	0.02	0.503	0.800	0.351
	*(Sampling 4)*	0.15	0.18	0.14	0.14	0.02			
**NEU (10^9^/L)**	*(Sampling 0)*	10.27	8.80	10.06	8.07	0.83	0.251	0.780	0.965
	*(Sampling 4)*	10.16	9.22	10.21	8.03	0.83			
**NLR**	*(Sampling 0)*	2.42	2.59	2.69	2.68	0.288	0.934	0.576	0.670
	*(Sampling 4)*	2.51	2.65	2.58	2.29	0.288			
**MLR**	*(Sampling 0)*	0.04	0.05	0.04	0.04	0.007	0.285	0.874	0.950
	*(Sampling 4)*	0.04	0.05	0.04	0.04	0.007			
**RBC (10^12^/L)**	*(Sampling 0)*	17.39	17.63	17.69	17.25	0.57	0.921	<0.0001	0.612
	*(Sampling 4)*	19.46	19.11	19.18	18.74	0.56			
**HGB (g/dL)**	*(Sampling 0)*	7.73 ^bc^	7.42 ^bc^	7.54 ^bc^	7.26 ^c^	0.264	0.416	<0.0001	0.817
	*(Sampling 4)*	8.73 ^a^	8.21 ^abc^	8.47 ^ab^	8.06 ^abc^	0.265			
**HCT (%)**	*(Sampling 0)*	24.75	24.45	24.47	23.83	0.677	0.627	<0.0001	0.849
	*(Sampling 4)*	26.73	25.81	26.09	25.31	0.682			
**MCV (fl)**	*(Sampling 0)*	14.72	14.05	14.01	13.84	0.414	0.779	0.015	0.572
	*(Sampling 4)*	13.67	13.68	13.61	13.53	0.418			
**MCH (pg)**	*(Sampling 0)*	4.59	4.20	4.27	4.18	0.131	0.166	0.408	0.670
	*(Sampling 4)*	4.48	4.30	4.42	4.31	0.132			
**MCHC (g/dL)**	*(Sampling 0)*	31.08	30.15	30.75	30.34	0.592	0.593	<0.0001	0.990
	*(Sampling 4)*	32.76	31.72	32.47	31.83	0.601			
**RDW-CV (%)**	*(Sampling 0)*	36.26	36.47	36.62	36.38	0.487	0.985	0.023	0.568
	*(Sampling 4)*	36.68	36.62	36.72	36.92	0.474			

PS_5: experimental diet with 5% of PS; PS_10: experimental diet with 10% of PS; PS_15: experimental diet with 15% of PS; SEM: Standard error of the mean; WBC: white blood cells; LYM: lymphocytes; MON: monocytes: NEU: neutrophils; NLR: neutrophils–lymphocytes ratio: MLR: monocytes–lymphocytes ratio; RBC: red blood cells; HGB: haemoglobin; HCT: haematocrit; MCV: mean corpuscular volume; MCHC: mean corpuscular haemoglobin concentration; RDW-CV: red blood cell distribution width-coefficient variation; ^a^, ^b^, ^c^: Different letters for the same variables indicate a significant difference between diet × sampling according to Tukey test (*p* < 0.05); n = 72 animals (18 goats/treatment, 2 batches/treatment).

**Table 4:** Effect of diets containing pomegranate silage on plasma biochemical profile of dairy goats.

Item		Diets					*p*-*Value*		
		Control	PS_5	PS_10	PS_15	SEM	Diet	Sampling	Diet × Sampling
**T4** **(nmol/L)**	*(Sampling 0)*	74.44 ^a^	74.03 ^ab^	75.06 ^ab^	77.51 ^ab^	2.30	0.741	<0.0001	0.716
	*(Sampling 4)*	81.50 ^b^	79.05 ^ab^	79.59 ^ab^	81.97 ^ab^	2.30			
**CHOL (mmol/L)**	*(Sampling 0)*	2.27	2.27	2.34	2.18	0.133	0.864	0.368	0.857
	*(Sampling 4)*	2.39	2.28	2.34	2.23	0.133			
**ALP** **(U/L)**	*(Sampling 0)*	407.17	701.00	606.32	285.42	160.7	0.462	0.057	0.117
	*(Sampling 4)*	432.72	601.68	484.21	284.24	157.4			
**ALT** **(U/L)**	*(Sampling 0)*	13.67 ^bc^	11.56 ^c^	12.42 ^c^	11.38 ^c^	0.854	0.239	<0.0001	0.127
	*(Sampling 4)*	18.39 ^a^	16.63 ^ab^	14.84 ^abc^	16.82 ^ab^	0.854			
**AMY** **(U/L)**	*(Sampling 0)*	106.67	49.69	64.05	58.52	19.26	0.449	0.649	0.099
	*(Sampling 4)*	88.22	61.78	64.31	56.81	19.26			
**TBIL (μmol/L)**	*(Sampling 0)*	4.11	4.22	4.11	4.11	0.177	0.510	0.032	0.509
	*(Sampling 4)*	4.11	4.42	4.63	4.50	0.177			
**BUN (mmol/L)**	*(Sampling 0)*	7.31 ^ab^	6.80 ^b^	7.21 ^ab^	6.70 ^b^	0.314	0.693	<0.0001	0.042
	*(Sampling 4)*	8.37 ^a^	8.31 ^a^	7.60 ^ab^	8.49 ^a^	0.314			
**CREA (μmol/L)**	*(Sampling 0)*	48.33 ^ab^	49.20 ^ab^	46.57 ^ab^	46.68 ^b^	3.631	0.257	0.002	0.075
	*(Sampling 4)*	55.17 ^ab^	57.36 ^ab^	44.52 ^b^	61.46 ^a^	3.629			
**Ca** **(mmol/L)**	*(Sampling 0)*	2.52	2.40	2.47	2.44	0.083	0.834	0.054	0.545
	*(Sampling 4)*	2.55	2.54	2.50	2.64	0.083			
**P** **(mmol/L)**	*(Sampling 0)*	1.65	1.80	1.96	1.82	0.162	0.698	0.011	0.921
	*(Sampling 4)*	1.56	1.61	1.76	1.67	0.162			
**GLU (mmol/L)**	*(Sampling 0)*	2.94 ^a^	3.17 ^ab^	3.06 ^abc^	3.41 ^abc^	0.120	0.717	<0.0001	0.384
	*(Sampling 4)*	3.49 ^bc^	3.64 ^c^	3.79 ^abc^	3.73 ^abc^	0.121			
**Na** **(mmol/L)**	*(Sampling 0)*	140.72 ^b^	138.54 ^c^	142.84 ^abc^	142.53 ^bc^	2.060	0.376	<0.0001	0.078
	*(Sampling 4)*	147.44 ^abc^	148.84 ^ab^	143.52 ^abc^	151.50 ^a^	2.060			
**K** **(mmol/L)**	*(Sampling 0)*	5.21	5.17	5.35	5.00	5.192	0.426	0.147	0.189
	*(Sampling 4)*	4.96	5.33	5.06	4.91	5.074			
**TP** **(g/L)**	*(Sampling 0)*	83.11 ^ab^	81.58 ^ab^	81.31 ^ab^	78.57 ^b^	2.214	0.615	<0.0001	0.278
	*(Sampling 4)*	88.61 ^a^	90.37 ^a^	85.27 ^ab^	89.98 ^a^	2.204			
**ALB** **(g/L)**	*(Sampling 0)*	40.72 ^b^	40.16 ^b^	40.74 ^b^	40.84 ^b^	1.207	0.329	<0.0001	0.008
	*(Sampling 4)*	45.27 ^ab^	44.54 ^ab^	40.63 ^b^	46.37 ^a^	1.207			
**GLOB (g/L)**	*(Sampling 0)*	42.61 ^ab^	41.00 ^ab^	40.57 ^ab^	37.84 ^b^	1.815	0.597	0.0001	0.304
	*(Sampling 4)*	43.33 ^ab^	46.00 ^a^	44.68 ^ab^	43.40 ^ab^	1.815			

PS_5: experimental diet with 5% of PS; PS_10: experimental diet with 10% of PS; PS_15: experimental diet with 15% of PS; SEM: Standard error of the mean; T4: thyroid hormone; CHOL: cholesterol; ALP: alkaline phosphatase; ALT: alanine aminotransferase; AMY: amylase; TBIL: total Bilirubin; BUN: blood urea nitrogen; CREA: creatinine; GLU: glucose; TP: total protein; ALB: albumin; GLOB; globulin; ^a^, ^b^, ^c^: Different letters for the same variables indicate a significant difference between diet × sampling according to Tukey test (*p* < 0.05); n = 72 animals (18 goats/treatment, 2 batches/treatment).

**Table 5:** Effect of diets containing pomegranate silage on plasma total and antioxidant capacity of dairy goats.

Item		Diets					*p*-*Value*		
		Control	PS_5	PS_10	PS_15	SEM	Diet	Sampling	Diet × Sampling
**ABTS ^1^**	*(Sampling 0)*	0.103 ^ab^	0.108 ^a^	0.098 ^ab^	0.100 ^ab^	0.004	0.607	<0.0001	0.607
	*(Sampling 4)*	0.090 ^ab^	0.089 ^b^	0.088 ^b^	0.087 ^b^	0.004			
**FRAP ^1^**	*(Sampling 0)*	0.544 ^bc^	0.558 ^bc^	0.520 ^c^	0.540 ^bc^	0.022	0.376	<0.0001	0.591
	*(Sampling 4)*	0.685 ^a^	0.684 ^a^	0.621 ^ab^	0.675 ^a^	0.022			
**SOD ^2^**	*(Sampling 0)*	0.338	0.332	0.323	0.319	0.028	0.950	0.330	0.474
	*(Sampling 4)*	0.311	0.353	0.356	0.353	0.028			

PS_5: experimental diet with 5% of PS; PS_10: experimental diet with 10% of PS; PS_15: experimental diet with 15% of PS; SEM: standard error of the mean; ABTS: 2,2′-azino-bis (3-ethylbenzothiazoline-6-sulfonate); FRAP: ferric reducing antioxidant power; SOD: superoxide dismutase activity; ^a^, ^b^, ^c^: Different letters for the same variables indicate a significant difference between diet × sampling according to Tukey test (*p* < 0.05); n = 72 animals (18 goats/treatment, 2 batches/treatment); ^1^ mg Trolox eq./mL. ^2^ U/mL.

## Data Availability

The original contributions presented in this study are included in the article. Further inquiries can be directed to the corresponding author.
